# The Complete Genome and Proteome of *Laribacter hongkongensis* Reveal Potential Mechanisms for Adaptations to Different Temperatures and Habitats

**DOI:** 10.1371/journal.pgen.1000416

**Published:** 2009-03-13

**Authors:** Patrick C. Y. Woo, Susanna K. P. Lau, Herman Tse, Jade L. L. Teng, Shirly O. T. Curreem, Alan K. L. Tsang, Rachel Y. Y. Fan, Gilman K. M. Wong, Yi Huang, Nicholas J. Loman, Lori A. S. Snyder, James J. Cai, Jian-Dong Huang, William Mak, Mark J. Pallen, Si Lok, Kwok-Yung Yuen

**Affiliations:** 1 State Key Laboratory of Emerging Infectious Diseases, The University of Hong Kong, Hong Kong, Special Administrative Region, People's Republic of China; 2 Research Centre of Infection and Immunology, The University of Hong Kong, Hong Kong, Special Administrative Region, People's Republic of China; 3 Department of Microbiology, The University of Hong Kong, Hong Kong, Special Administrative Region, People's Republic of China; 4 Centre for Systems Biology, University of Birmingham, Birmingham, United Kingdom; 5 Department of Biochemistry, The University of Hong Kong, Hong Kong, Special Administrative Region, People's Republic of China; 6 Genome Research Centre, The University of Hong Kong, Hong Kong, Special Administrative Region, People's Republic of China; Progentech, United States of America

## Abstract

*Laribacter hongkongensis* is a newly discovered Gram-negative bacillus of the *Neisseriaceae* family associated with freshwater fish–borne gastroenteritis and traveler's diarrhea. The complete genome sequence of *L. hongkongensis* HLHK9, recovered from an immunocompetent patient with severe gastroenteritis, consists of a 3,169-kb chromosome with G+C content of 62.35%. Genome analysis reveals different mechanisms potentially important for its adaptation to diverse habitats of human and freshwater fish intestines and freshwater environments. The gene contents support its phenotypic properties and suggest that amino acids and fatty acids can be used as carbon sources. The extensive variety of transporters, including multidrug efflux and heavy metal transporters as well as genes involved in chemotaxis, may enable *L. hongkongensis* to survive in different environmental niches. Genes encoding urease, bile salts efflux pump, adhesin, catalase, superoxide dismutase, and other putative virulence factors—such as hemolysins, RTX toxins, patatin-like proteins, phospholipase A1, and collagenases—are present. Proteomes of *L. hongkongensis* HLHK9 cultured at 37°C (human body temperature) and 20°C (freshwater habitat temperature) showed differential gene expression, including two homologous copies of *argB*, *argB*-20, and *argB*-37, which encode two isoenzymes of *N*-acetyl-L-glutamate kinase (NAGK)—NAGK-20 and NAGK-37—in the arginine biosynthesis pathway. NAGK-20 showed higher expression at 20°C, whereas NAGK-37 showed higher expression at 37°C. NAGK-20 also had a lower optimal temperature for enzymatic activities and was inhibited by arginine probably as negative-feedback control. Similar duplicated copies of *argB* are also observed in bacteria from hot springs such as *Thermus thermophilus*, *Deinococcus geothermalis*, *Deinococcus radiodurans*, and *Roseiflexus castenholzii*, suggesting that similar mechanisms for temperature adaptation may be employed by other bacteria. Genome and proteome analysis of *L. hongkongensis* revealed novel mechanisms for adaptations to survival at different temperatures and habitats.

## Author Summary

*Laribacter hongkongensis* is a recently discovered bacterium associated with gastroenteritis and traveler's diarrhea. Freshwater fish is the reservoir of *L. hongkongensis*. In order to achieve a rapid understanding on the mechanisms by which the bacterium adapts to different habitats and its potential virulence factors, we sequenced the complete genome of *L. hongkongensis*, compared its gene contents with other bacteria, and compared its gene expression at 37°C (human body temperature) and 20°C (freshwater habitat temperature). We found that the gene contents of *L. hongkongensis* enable it to adapt to its diverse habitats of human and freshwater fish intestines and freshwater environments. Genes encoding proteins responsible for survival in the intestinal environments, adhesion to intestinal cells, evasion from host immune systems, and putative virulence factors similar to those observed in other pathogens are present. We also observed, in gene expression studies, that *L. hongkongensis* may be using different pathways for arginine synthesis regulated at different temperatures. Phylogenetic analysis suggested that such mechanisms for temperature adaptation may also be used in bacteria found in extreme temperatures.

## Introduction

*Laribacter hongkongensis* is a recently discovered, Gram-negative, facultative anaerobic, motile, seagull or S-shaped, asaccharolytic, urease-positive bacillus that belongs to the *Neisseriaceae* family of β-proteobacteria [1]. It was first isolated from the blood and thoracic empyema of an alcoholic liver cirrhosis patient in Hong Kong [2]. In a prospective study, *L. hongkongensis* was shown to be associated with community acquired gastroenteritis and traveler's diarrhea [3],[4]. *L. hongkongensis* is likely to be globally distributed, as travel histories from patients suggested its presence in at least four continents: Asia, Europe, Africa and Central America [4]–[6]. *L. hongkongensis* has been found in up to 60% of the intestines of commonly consumed freshwater fish, such as grass carp and bighead carp [4],[7],[8]. It has also been isolated from drinking water reservoirs in Hong Kong [9]. Pulsed-field gel electrophoresis and multilocus sequence typing showed that the fish and patient isolates fell into separate clusters, suggesting that some clones could be more virulent or adapted to human [8],[10]. These data strongly suggest that this bacterium is a potential diarrheal pathogen that warrants further investigations.

Compared to other families such as *Enterobacteriaceae*, *Vibrionaceae*, *Streptococcaceae*, genomes of bacteria in the *Neisseriaceae* family have been relatively under-studied. Within this family, *Neisseria meningitidis*, *Neisseria gonorrhoeae* and *Chromobacterium violaceum* are the only species with completely sequenced genomes [11]–[13]. In view of its potential clinical importance, distinct phylogenetic position, interesting phenotypic characteristics and the availability of genetic manipulation systems [14]–[17], we sequenced and annotated the complete genome of a strain (HLHK9) of *L. hongkongensis* recovered from a 36-year old previously healthy Chinese patient with profuse diarrhea, vomiting and abdominal pain [4]. Proteomes of *L. hongkongensis* growing at 37°C (body temperature of human) and 20°C (average temperature of freshwater habitat in fall and winter) [9] were also compared.

## Results/Discussion

### General Features of the Genome

**Figure 1:**
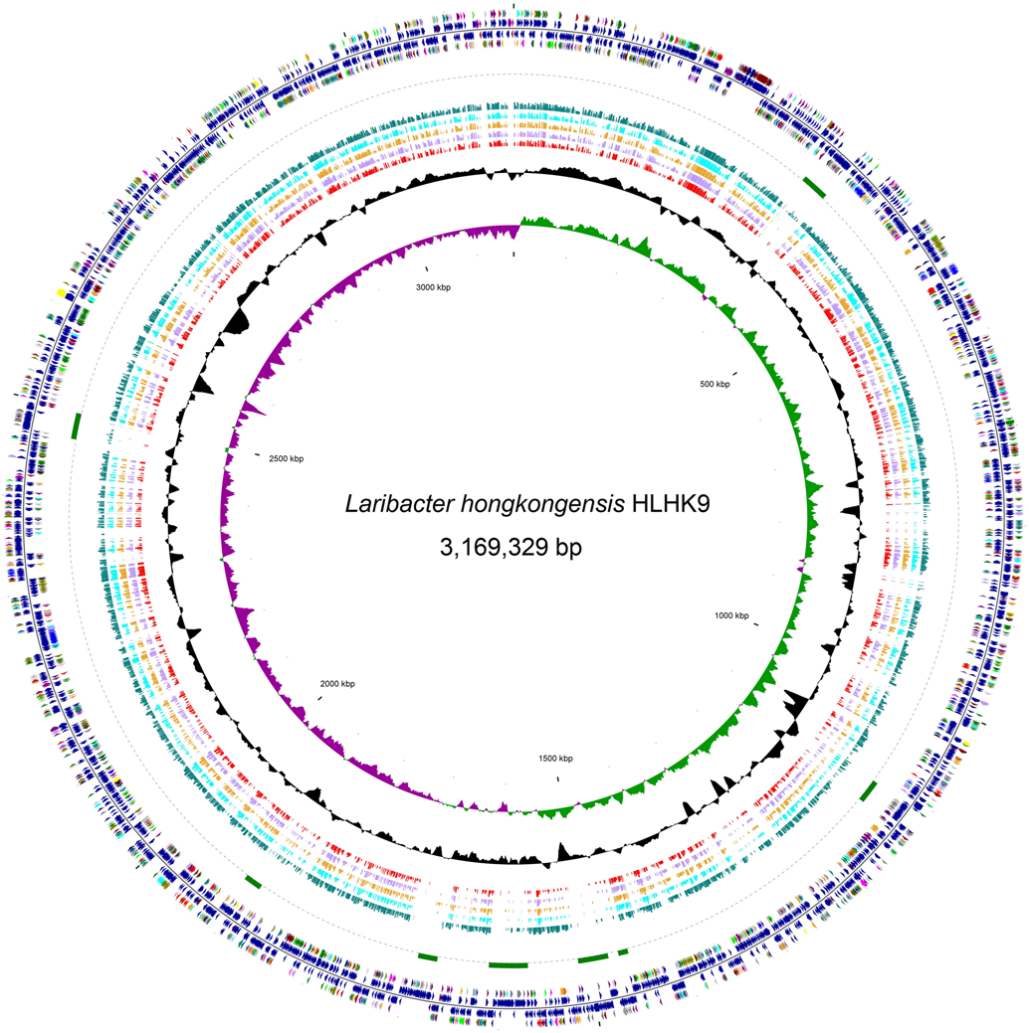
Circular representation of the genome of *L. hongkongensis* HLHK9. From the inside: circles 1 and 2, GC skew (dark green indicates values >0 and dark purple indicates values <0) and G+C content (10-kb window with 100-b step); circles 3 to 7, red, light purple, orange, aqua and teal bars show BLAST hits to *Neisseria gonorrhoeae* FA 1090, *Neisseria gonorrhoeae* MC58, *Neisseria gonorrhoeae* FAM18, *Neisseria gonorrhoeae* Z2491 and *Chromobacterium violaceum* ATCC 12472, respectively; circle 8, green arcs show location of eight putative prophages; circles 9 and 12, colors reflect Cluster of Orthologous Groups of coding sequences (CDSs). Maroon, translation, ribosomal structure and biogenesis; navy, transcription; purple, DNA replication, recombination and repair; light brown, cell division and chromosome partitioning; aqua, posttranslational modification, protein turnover, chaperones; teal, cell envelope biogenesis, outer membrane; blue, cell motility and secretion; orange, inorganic ion transport and metabolism; light purple, signal transduction mechanisms; olive, energy production and conversion; lime, carbohydrate transport and metabolism; green, amino acid transport and metabolism; fuchsia, nucleotide transport and metabolism; light pink, coenzyme metabolism; red, lipid metabolism; yellow, secondary metabolites biosynthesis, transport and catabolism; gray, general function prediction only; silver, function unknown; circles 10 and 11, dark blue, dark red and dark purple indicate CDSs, tRNA and rRNA on the − and + strands, respectively.

**Figure 2:**
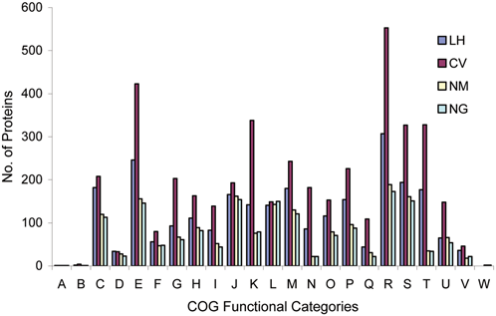
Coding sequence function distribution in genomes of *L. hongkongensis*, *C. violaceum*, *N. meningitidis* and *N. gonorrhoeae*. The columns indicate the number of proteins in different Cluster of Orthologous Groups (COG) functional categories (www.ncbi.nlm.nih.gov/COG). LH, *L. hongkongensis*; CV, *C. violaceum*; NM, *N. meningitidis*, NG, *N. gonorrhoeae*.

The complete genome of *L. hongkongensis* is a single circular chromosome of 3,169,329 bp with a G+C content of 62.35% (Figure 1). In terms of genome size and number of predicted coding sequences (CDSs), rRNA operons and tRNA genes (Table 1), *L. hongkongensis* falls into a position intermediate between *C. violaceum* and the pathogenic *Neisseria* species. A similar intermediate status was also observed when the CDSs were classified into Cluster of Orthologous Groups (COG) functional categories, except for genes of RNA processing and modification (COG A), cell cycle control, mitosis and meiosis (COG D), replication, recombination and repair (COG L) and extracellular structures (COG W), of which all four bacteria have similar number of genes (Figure 2). This is in line with the life cycles and growth requirements of the bacteria. *C. violaceum* is a highly versatile, facultative anaerobic, soil- and water-borne free-living bacterium and therefore requires the largest genome size and gene number. The pathogenic *Neisseria* species are strictly aerobic bacteria with human as the only host and therefore require the smallest genome size and gene number. *L. hongkongensis* is a facultative anaerobic bacterium that can survive in human, freshwater fish and 0–2% NaCl but not in marine fish or ≥3% NaCl and therefore requires an intermediate genome size and gene number.

**Table 1:** Comparison of general features of the genomes of *L. hongkongensis* (*LH*), *C. violaceum* (*CV*), *N. meningitidis* (*NM*) and *N. gonorrhoeae* (*NG*).

Features	*LH* HLHK9	*CV* ATCC12472	*NG* NCCP11945	*NM* MC58
Size, bp	3,169,329	4,751,080	2,232,025	2,272,351
G+C content	62.35	64.83	52.4	51.5
No. of CDSs	3,237	4,431	2,662	2,158
% of genome constituting coding regions	88	89	87	83
No. of rRNA operons	7	8	4	4
No. of tRNA	78	98	54	59

Data of *CV*, *NG* and *NM* were from [ref. 11],[12] and [13].

### General Metabolism

The *L. hongkongensis* genome lacks a complete set of enzymes for glycolysis, with orthologues of glucokinase, 6-phosphofructokinase and pyruvate kinase being absent (Table S1). This is compatible with its asaccharolytic phenotype and is consistent with other asaccharolytic bacteria, such as *Campylobacter jejuni*, *Bordetella pertussis*, *Bordetella parapertussis* and *Bordetella bronchiseptica*, in that glucokinase and 6-phosphofructokinase are also absent from their genomes [18],[19]. On the other hand, the *L. hongkongensis* genome encodes the complete sets of enzymes for gluconeogenesis, the pentose phosphate pathway and the glyoxylate cycle (Table S1). Similar to *C. jejuni*, the *L. hongkongensis* genome encodes a number of extracellular proteases and amino acid transporters. These amino acids can be used as carbon source for the bacterium. The genome encodes enzymes for biosynthesis of the 21 genetically encoded amino acids and for biosynthesis and β-oxidation of saturated fatty acids (Tables S2 and S3). The *L. hongkongensis* genome encodes a variety of dehydrogenases (LHK_00527–00540, LHK_01219–01224, LHK_02418–02421, LHK_00801–00803, LHK_01861, LHK_02912–02913 and LHK_00934) that enable it to utilize a variety of substrates as electron donors, such as NADH, succinate, formate, proline, acyl-CoA and D-amino acids. The presence of three terminal cytochrome oxidases may allow *L. hongkongensis* to carry out respiration using oxygen as the electron acceptor under both aerobic conditions [type aa_3_ oxidase (LHK_00169–00170, LHK_00173)] and conditions with reduced oxygen tension [type cbb_3_ (LHK_00995–00996, LHK_00998) and type bd (LHK_02252–02253) oxidases]. The genome also encodes a number of reductases [fumarate reductase (LHK_02340–02342), nitrate reductase (LHK_02079–02085), dimethylsulfoxide (DMSO) reductase (LHK_02496–02498) and tetrathionate reductase (LHK_01476–01478)], which may help carry out respiration with alternative electron acceptors to oxygen (fumarate, nitrate, DMSO and tetrathionate) under anaerobic conditions. This is supported by the enhanced growth of *L. hongkongensis* under anaerobic conditions in the presence of nitrate (data not shown). Further studies are required to confirm if the bacterium can utilize other potential electron acceptors.

### Transporters

There were 441 transport-related proteins (13.6% of all CDSs) in the *L. hongkongensis* genome, comprising an extensive variety of transporters, which may reflect its ability to adapt to the freshwater fish and human intestines, and freshwater environments. According to the Transporter Classification Database (TCDB) (http://www.tcdb.org/), all seven major categories of transporters are present in *L. hongkongensis*. Primary active transporters (class 3 transporters) were the most abundant class of transporters, accounting for 43.3% (191 CDSs) of all annotated CDSs related to transport, among which 104 belong to the ATP-binding cassette (ABC) transporter superfamily and 41 were oxidoreduction-driven transporters. Electrochemical potential-driven transporters (class 2 transporters) were the second most abundant class of transporters, accounting for 27.9% (123 CDSs) of all annotated CDSs related to transport, most of which (117 CDSs) are various kinds of porters including major facilitator superfamily (MFS) (19 CDSs), resistance-nodulation-cell division (RND) superfamily (22 CDSs), amino acid-polyamine-organocation family (8 CDSs), dicarboxylate/amino acid∶cation symporter (DAACS) family (5 CDSs) and monovalent cation∶proton antiporter-2 family (3 CDSs), and various heavy metal transporters which may be involved in detoxification and resistance against environmental hazards. Three different types of class 2 transporters, belonging to the DAACS, tripartite ATP-independent periplasmic transporter and C_4_-dicarboxylate uptake C family, are likely involved in the transport of malate, which can be used as the sole carbon source for *L. hongkongensis* in minimal medium [unpublished data]. The remaining class 2 transporters were ion-gradient-driven energizers belonging to the TonB family (6 CDSs). The third most abundant class of transporters was the channels and pores (class 1), with 39 CDSs including 12 α-type channels, 26 β-barrel porins. Among the 12 α-type channels, four were mechanosensitive channels which are important for mediating resistance to mechanophysical changes. The remaining transporters belong to four other classes, namely group translocators (class 4, 9 CDSs), transport electron carriers (class 5, 16 CDSs), accessory factors involved in transport (class 8, 9 CDSs) and incompletely characterized transport system (class 9, 54 CDSs). In line with their asaccharolytic nature, the genomes of *L. hongkongensis* and *C. jejuni* do not contain genes that encode a complete phosphotransferase system. The five families of multidrug efflux transporters, including MFS (6 CDSs), RND (8 CDSs), small multidrug resistance family (2 CDSs), multidrug and toxic compound extrusion family (2 CDSs) and ABC transporter superfamily (5 CDSs), were all present in *L. hongkongensis*, which may reflect its ability to withstand toxic substances in different habitats [20]. 20 CDSs were related to iron metabolism, including hemin transporters, ABC transporters of the metal type and ferrous iron, iron-storage proteins and the Fur protein responsible for iron uptake regulation. In contrast to *C. violaceum* which produces siderophores for iron acquisition, but similar to the pathogenic *Neisseria* species, proteins related to siderophore formation are not found in *L. hongkongensis* genome. In addition to a TonB-dependent siderophore receptor (LHK_00497), a set of genes (LHK_01190, LHK_01193, LHK_01427–1428) related to the transport of hemin were present, suggesting that *L. hongkongensis* is able to utilize exogenous siderophores or host proteins for iron acquisition, which may be important for survival in different environments and hosts.

### Motility, Chemotaxis, and Signal Transduction

Except the first strain of *L. hongkongensis* isolated from the blood and empyema pus of a patient which represented a non-motile variant, all *L. hongkongensis* strains, whether from human diarrheal stool, fish intestine or environmental water, are motile with polar flagella. The ability to sense and respond to environmental signals is important for survival in changing ecological niches. A total of 47 CDSs are related to chemotaxis, of which 27 encode methyl-accepting chemotaxis proteins (MCPs) and 20 encode chemosensory transducer proteins. While most MCPs are scattered throughout the genome, the transducer proteins are mostly arranged in three gene clusters (Figure S1). At least 38 genes, in six gene clusters, are involved in the biosynthesis of flagella (Figure S2).

Enteric bacteria use several quorum-sensing mechanisms, including the LuxR-I, LuxS/AI-2, and AI-3/epinephrine/norepinephrine systems, to recognize the host environment and communicate across species. Unlike the genomes of *C. violaceum* and the pathogenic *Neisseria* species which encode genes involved in LuxR-I and LuxS/AI-2 systems respectively, the *L. hongkongensis* genome does not encode genes of these 2 systems. Instead, the AI-3/epinephrine/norepinephrine system, which is involved in inter-kingdom cross-signaling and regulation of virulence gene transcription and motility, best characterized in enterohemorrhagic *E. coli* [21],[22], is likely the predominant quorum-sensing mechanism used by *L. hongkongensis*. Several human enteric commensals or pathogens, including *E. coli*, *Shigella*, and *Salmonella*, produce AI-3 [23]. A two-component system, QseB/C, of which QseC is the sensor kinase and QseB the response regulator, has been found to be involved in sensing AI-3 from bacteria and epinephrine/norepinephrine from host, and activation of the flagellar regulon transcription [21]. While the biosynthetic pathway of AI-3 has not been discovered, two sets of genes, LHK_00329/LHK_00328 and LHK_01812/LHK_01813, homologous to QseB/QseC were identified in the *L. hongkongensis* genome, suggesting that the bacterium may regulate its motility upon recognition of its host environment. The presence of two sets of QseB/QseC, one most similar to those of *C. violaceum* and the other most homologous to *Azoarcus* sp. strain BH72, is intriguing, as the latter is the only bacterium, with complete genome sequence available, that possesses two copies of such genes.

### Pathogenic Factors

Before reaching the human intestine, *L. hongkongensis* has to pass through the highly acidic environment of the stomach. In the *L. hongkongensis* genome, a cluster of genes, spanning a 12-kb region, related to acid resistance, is present. Similar to *Helicobacter pylori*, the *L. hongkongensis* genome contains a complete urease gene cluster (LHK_01035–LHK_01037, LHK_01040–LHK_01044), in line with the bacterium's urease activity. Phylogenetically, all 8 genes in the urease cassette are most closely related to the corresponding homologues in *Brucella* species (α-proteobacteria), *Yersinia* species (γ-proteobacteria) and *Photorhabdus luminescens* (γ-proteobacteria), instead of those in other members of β-proteobacteria, indicating that *L. hongkongensis* has probably acquired the genes through horizontal gene transfer after its evolution into a distinct species (Figure S3). Upstream and downstream to the urease cassette, *adi* (LHK_01034) and *hdeA* (LHK_01046) were found respectively. Their activities will raise the cytoplasmic pH and prevents proteins in the periplasmic space from aggregation during acid shock respectively [24],[25]. In addition to the acid resistance gene cluster, the *L. hongkongensis* genome contains two *arc* gene clusters [*arcA* (LHK_02729 and LHK_02734), *arcB* (LHK_02728 and LHK_02733), *arcC* (LHK_02727 and LHK_02732) and *arcD* (LHK_02730 and LHK_02731)] of the arginine deiminase pathway which converts L-arginine to carbon dioxide, ATP, and ammonia. The production of ammonia increases the pH of the local environment [26],[27].

Similar to other pathogenic bacteria of the gastrointestinal tract, the genome of *L. hongkongensis* encodes genes for bile resistance. These include three complete copies of *acrAB* (LHK_01425–01426, LHK_02129–02130 and LHK_02929–02930), encoding the best studied efflux pump for bile salts, and two pairs of genes (LHK_01373–01374 and LHK_03132–03133) that encode putative efflux pumps homologous to that encoded by *emrAB* in *E. coli* [28]. Furthermore, five genes [*tolQ* (LHK_00053), *tolR* (LHK_03174), *tolA* (LHK_03173), *tolB* (LHK_03172) and *pal* (LHK_03171)] that encode the Tol proteins, important in maintaining the integrity of the outer membrane and for bile resistance, are also present [29].

In the *L. hongkongensis* genome, a putative adhesin (LHK_01901) for colonization of the intestinal mucosa, most closely related to the adhesins of diffusely adherent *E. coli* (DAEC) and enterotoxigenic *E. coli* (ETEC), encoded by *aidA* and *tibA* respectively, was observed (Figure S4) [30],[31]. *aidA* and *tibA* encode proteins of the autotransporter family, type V protein secretion system of Gram-negative bacteria. All the three domains (an N-terminal signal sequence, a passenger domain and a translocation domain) present in proteins of this family are found in the putative adhesin in *L. hongkongensis*. Moreover, a putative heptosyltransferase (LHK_01902), with 52% amino acid identity to the TibC heptosyltransferase of ETEC, responsible for addition of heptose to the passenger domain, was present upstream to the putative adhesin gene in the *L. hongkongensis* genome (Figure S4). In addition to host cell adhesion, the passenger domains of autotransporters may also confer various virulence functions, including autoaggregation, invasion, biofilm formation and cytotoxicity. The *L. hongkongensis* genome encodes a putative superoxide dismutase (LHK_01716) and catalases (LHK_01264, LHK_01300 and LHK_02436), which may play a role in resistance to superoxide radicals and hydrogen peroxide generated by neutrophils.

The same set of genes that encode enzymes for synthesis of lipid A (endotoxin), the two Kdo units and the heptose units of lipopolysaccharide (LPS) are present in the genomes of *L. hongkongensis*, *C. violaceum*, *N. meningitidis*, *N. gonorrhoeae* and *E. coli*. Moreover, 9 genes [*rfbA* (LHK_02995), *rfbB* (LHK_02997), *rfbC* (LHK_02994), *rfbD* (LHK_02996), *wbmF* (LHK_02799), *wbmG* (LHK_02800), *wbmH* (LHK_02801), *wbmI* (LHK_02790) and *wbmK* (LHK_02792)] that encode putative enzymes for biosynthesis of the polysaccharide side chains are present in the *L. hongkongensis* genome. In addition to genes for synthesizing LPS, a number of CDSs that encode putative cytotoxins are present, including cytotoxins that act on the cell surface [hemolysins (LHK_00956 and LHK_03166) and RTX toxins (LHK_02735 and LHK_02918)] and those that act intracellularly [patatin-like proteins (LHK_00116, LHK_01938, and LHK_03113)] [32],[33]. Furthermore, a number of CDSs that encode putative outer membrane phospholipase A1 (LHK_00790) and collagenases (LHK_00305–00306, LHK_00451, and LHK_02651) for possible bacterial invasion are present.

### Adaptability to Different Environmental Temperatures

**Figure 3:**
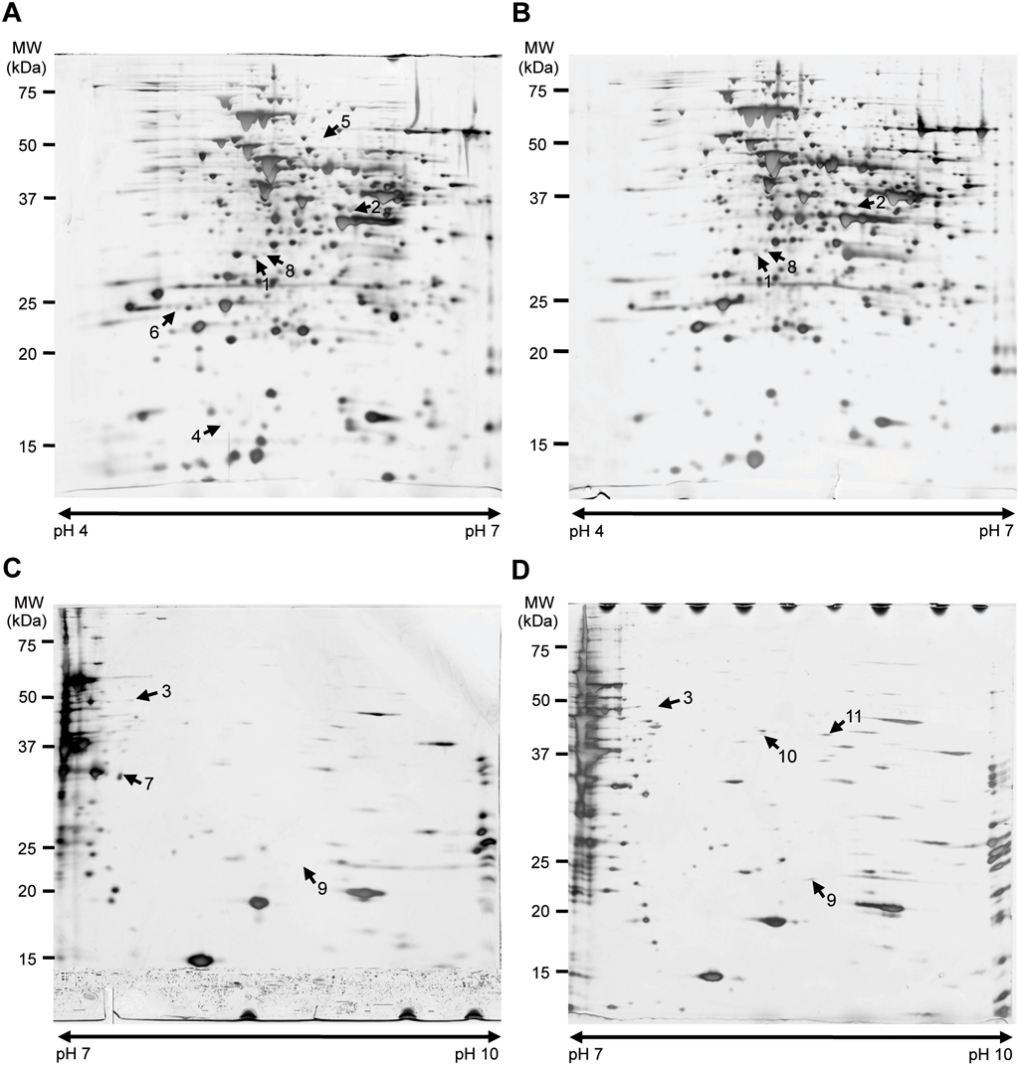
2D gel electrophoresis patterns of proteins extracted from *L. hongkongensis* HLHK9 cells grown at 20°C and 37°C. Comparison of soluble proteins from *L. hongkongensis* HLHK9 grown at (A) 20°C and (B) 37°C in the pH range of 4–7 and (C) 20°C and (D) 37°C in the pH range of 7–10. The arrowheads indicate spots with differential expression. Molecular masses (MW) and pH are indicated.

**Figure 4:**
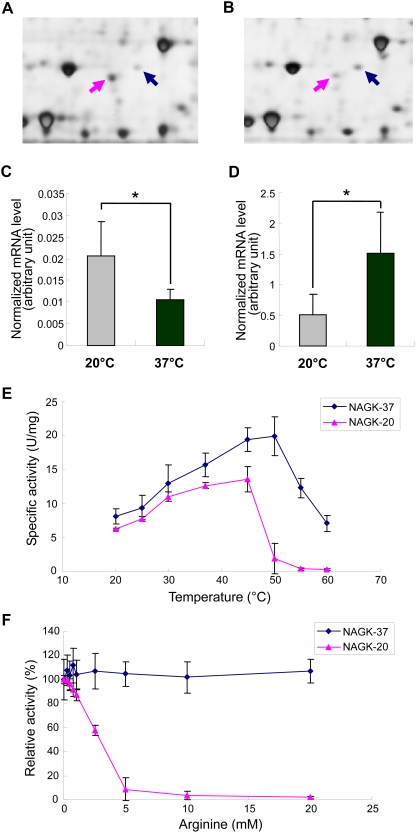
Proteomics, transcriptional and biochemical analysis of *N*-acetyl-L-glutamate kinase (NAGK)-20 and NAGK-37. Differential expressions of NAGK-20 (pink arrow) and NAGK-37 (blue arrow) in *L. hongkongensis* cultured at 20°C (A) and 37°C (B). Normalized mRNA levels of *argB*-20 (C) and *argB*-37 (D) in *L. hongkongensis* cells cultured at 20°C and 37°C. Data were analyzed by unpaired Student's t-test. The data represent the means of three independent experiments. Error bars represent standard deviations. Significant changes are represented by asterisks (*, *P*<0.05). (E) Specific kinase activities of purified NAGK-20 (pink) and NAGK-37 (blue) measured at different temperatures (25–60°C). The data represent the means of three independent experiments. Error bars represent standard deviations. (F) Effects of arginine on kinase activities of purified NAGK-20 (pink) and NAGK-37 (blue) measured in arginine at different concentrations (0.25–20 mM). The data represent the means of three independent experiments. Error bars represent standard deviations.

To better understand how *L. hongkongensis* adapts to human body and freshwater habitat temperatures at the molecular level, the types and quantities of proteins expressed in *L. hongkongensis* HLHK9 cultured at 37°C and 20°C were compared. Since initial 2D gel electrophoresis analysis of *L. hongkongensis* HLHK9 proteins under a broad range of p*I* and molecular weight conditions revealed that the majority of the proteins reside on the weakly acidic to neutral portion, with a minority on the weak basic portion, consistent with the median p*I* value of 6.63 calculated for all putative proteins in the genome of *L. hongkongensis* HLHK9, we therefore focused on IPG strips of pH 4–7 and 7–10. Comparison of the 2D gel electrophoresis patterns from *L. hongkongensis* HLHK9 cells grown at 20°C and 37°C revealed 12 differentially expressed protein spots, with 7 being more highly expressed at 20°C than at 37°C and 5 being more highly expressed at 37°C than at 20°C (Table 2, Figure 3). The identified proteins were involved in various functions (Table 2). Of note, spot 8 [*N*-acetyl-L-glutamate kinase (NAGK)-37, encoded by *argB-37*] was up-regulated at 37°C, whereas spot 1 (NAGK-20, encoded by *argB-20*), was up-regulated at 20°C (Figures 3, 4A and 4B). These two homologous copies of *argB* encode two isoenzymes of NAGK [NAGK-20 (LHK_02829) and NAGK-37 (LHK_02337)], which catalyze the second step of the arginine biosynthesis pathway.

**Table 2:** Differential protein expression of *L. hongkongensis* at 20°C and 37°C.

Spot no.	Gene no.	Theoretical MW (kDa)	Theoretical p*I*	Proteins	Functional categories (COG)	Sequence coverage (%)	Peptide matched	MOWSE score	20°C/37°C ratio^a^	*p* value
Higher expression at 20°C										
1	LHK_02829	30.0	5.03	Acetylglutamate kinase, NAGK-20	Amino acid transport and metabolism	24	6	9.49e+003	+2.50	<0.005
2	LHK_01819	39.0	6.30	Probable porin protein	Cell wall/membrane/envelope biogenesis	36	9	3.86e+005	+2.41	<0.005
3	LHK_00236	49.5	7.06	Peptidase M16 domain protein precursor	—	33	14	2.47e+005	+2.04	<0.05
Only at 20°C										
4	LHK_00011	16.9	4.99	Protein-L-isoaspartate (D-aspartate) O-methyltransferase	Post-translational modification, protein turnover, chaperones	41	9	6.28e+03	—	<0.01
5	LHK_01152	49.0	5.44	Probable phage sheath protein	—	28	10	1.11e+005	—	<0.05
6	LHK_01472	23.4	4.70	Ribonuclease activity regulator protein RraA	General function prediction only	36	7	4.13e+03	—	<0.01
7	LHK_01018	30.3	6.66	Methylenetetrahydro-folate dehydrogenase/cyclohydrolase	Coenzyme transport and metabolism	45	14	6.67e+007	—	<0.05
Higher expression at 37°C										
8	LHK_02337	31.5	5.12	Acetylglutamate kinase, NAGK-37	Amino acid transport and metabolism	38	13	3.48e+004	−2.09	<0.005
9	LHK_02119	23.7	9.22	Hypothetical protein	—	41	8	1.24e+004	−3.06	<0.005
Only at 37°C										
10	LHK_02507	44.6	9.11	Probable *N*-acetylmuramoyl-L-alanine amidase	Cell wall/membrane/envelope biogenesis	58	20	6.01e+009	—	<0.001
11	LHK_02507	44.6	9.11	Probable *N*-acetylmuramoyl-L-alanine amidase	Cell wall/membrane/envelope biogenesis	68	23	7.6e+010	—	<0.05
12	LHK_03194	48.9	7.94	Survival protein SurA precursor	Posttranslational modification, protein turnover, chaperones	33	10	3.79e+006	—	<0.005

a A negative number indicates the inverse ratio of that indicated in the column heading.

The transcription levels of *argB-20* and *argB-37* at 20°C and 37°C were quantified by real time RT-PCR. Results showed that the mRNA level of *argB-20* at 20°C was significantly higher that at 37°C and the mRNA level of *argB-37* at 37°C was significantly higher that at 20°C (Figure 4C and 4D), suggesting that their expressions, similar to most other bacterial genes, were controlled at the transcription level. When *argB-20* and *argB-37* were cloned, expressed and the corresponding proteins NAGK-20 and NAGK-37 purified for enzyme assays, their highest enzymatic activities were observed at 37–45°C and 45–50°C respectively (Figure 4E). Moreover, NAGK-20, but not NAGK-37, was inhibited by 0.25–10 mM of arginine (Figure 4F).

*L. hongkongensis* probably regulates arginine biosynthesis at temperatures of different habitats using two pathways with two isoenzymes of NAGK. *L. hongkongensis* and wild type *E. coli* ATCC 25922, but not *E. coli* JW5553-1 (*argB* deletion mutant), grew in minimal medium without arginine, indicating that *L. hongkongensis* contains a functional arginine biosynthesis pathway. NAGK-20 is expressed at higher level at 20°C than 37°C, whereas NAGK-37 is expressed at higher level at 37°C than 20°C. Bacteria use either of two different pathways, linear and cyclic, for arginine biosynthesis. Similar to NAGK-20 of *L. hongkongensis*, NAGK of *Pseudomonas aeruginosa* and *Thermotoga maritima*, which employ the cyclic pathway, can be inhibited by arginine as the rate-limiting enzyme for negative feedback control [34]–[37]. On the other hand, similar to NAGK-37 of *L. hongkongensis*, NAGK of *E. coli*, which employs the linear pathway, is not inhibited by arginine [35],[36]. We speculate that *L. hongkongensis* can use different pathways with the two NAGK isoenzymes with differential importance at different temperatures of different habitats.

**Figure 5:**
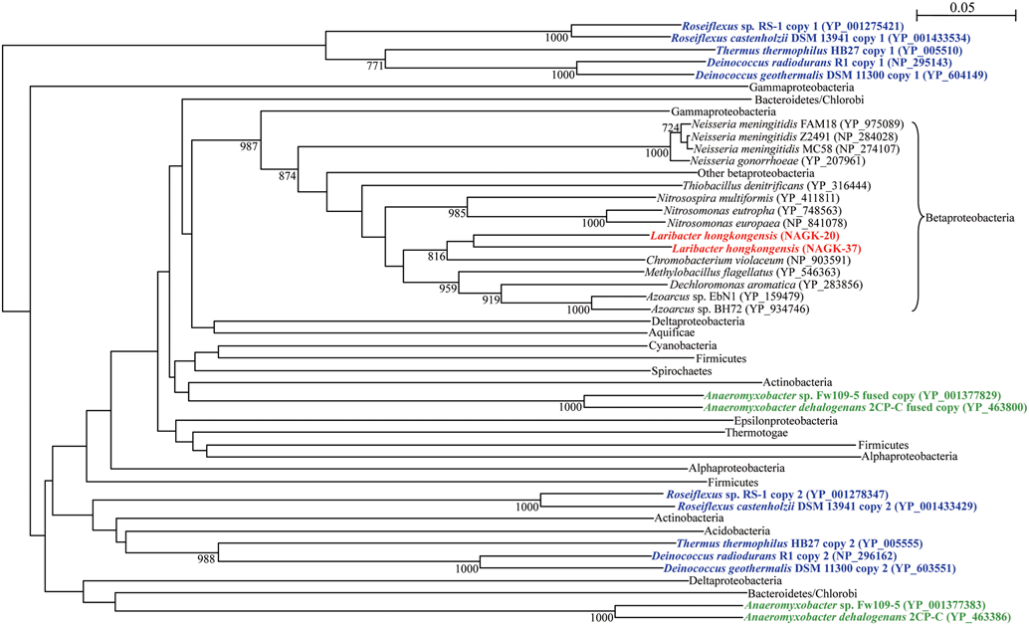
Phylogenetic relationships among *N*-acetyl-L-glutamate kinase (NAGK)-20 and NAGK-37 of *L. hongkongensis* and their homologues in other bacteria. NAGK-20 and NAGK-37 of *L. hongkongensis* were highlighted in red. *Thermus thermophilus*, *Deinococcus geothermalis*, *Deinococcus radiodurans*, *Roseiflexus castenholzii* and *Roseiflexus* sp. RS-1 with two distinct copies of *argB* were highlighted in blue, whereas *Anaeromyxobacter* sp. Fw109-5 and *Anaeromyxobacter dehalogenans* 2CP-C with one distinct copy of *argB* and one copy of *argJ* fused with *argB* were highlighted in green. Bootstrap values were calculated from 1,000 trees. The scale bar indicates the estimated number of substitutions per 20 amino acids. All names and accession numbers are given as cited in the GenBank database.

Phylogenetic analysis of NAGK-20 and NAGK-37 showed that they were more closely related to each other than to homologues in other bacteria (Figure 5). The topology of the phylogenetic tree constructed using NAGK was similar to that constructed using 16S rRNA gene sequences (data not shown). This suggested that the evolution of *argB* genes in general paralleled the evolution of the corresponding bacteria, and *argB* gene duplication has probably occurred after the evolution of *L. hongkongensis* into a separate species. The requirement to adapt to different temperatures and habitats may have provided the driving force for subsequent evolution to 2 homologous proteins that serve in different environments. Notably, among all 465 bacterial species with complete genome sequences available, only *Thermus thermophilus*, *Deinococcus geothermalis*, *Deinococcus radiodurans*, *Roseiflexus castenholzii* and *Roseiflexus* sp. RS-1 possessed two copies of *argB*, whereas *Anaeromyxobacter* sp. Fw109-5 and *Anaeromyxobacter dehalogenans* 2CP-C possessed one copy of *argB* and another fused with *argJ* (Figure 5). The clustering of *argB* in two separate groups in these bacteria suggests that *argB* gene duplication has probably occurred in their ancestor, before the divergence into separate species. The prevalence of *T. thermophilus*, *Deinococcus* species and *Roseiflexus* species in hot springs suggested that this novel mechanism of temperature adaptation may also be important for survival at different temperatures in other bacteria. Further experiments on differential expression of the two isoenzymes at different temperatures in these bacteria will verify our speculations.

### Conclusion

Traditionally, complete genomes of bacteria with medical, biological, phylogenetic or industrial interests were sequenced only after profound phenotypic and genotypic characterization of the bacteria had been performed. With the advance in technology and bioinformatics tools, complete genome sequences of bacteria can be obtained with greater ease. In this study, we sequenced and analyzed the complete genome of *L. hongkongensis*, a newly discovered bacterium of emerging medical and phylogenetic interest, and performed differential proteomics and downstream characterization of important pathways. In addition, putative virulence factors and a putative novel mechanism of arginine biosynthesis regulation at different temperatures were discovered, further characterization of which will lead to better understanding of their contributions to the survival and virulence of *L. hongkongensis*, the *Neisseriaceae* family and other bacteria. A similar “reverse genomics” approach can be used for the study of other newly discovered important bacteria.

## Materials and Methods

### Sequencing and Assembly

The genome sequence of *L. hongkongensis* HLHK9 was determined with the whole-genome shotgun method. Three shotgun libraries were generated: one small-insert (2–4 kb) library and one medium-insert (5–6 kb) library in pcDNA2.1, and a large-insert (35–45 kb) fosmid library in pCC2FOS. DNA sequencing was performed using dye-terminator chemistries on ABI3700 sequencers. Shotgun sequences were assembled with Phrap. Fosmid end sequences were mapped onto the assembly using BACCardI [38] for validation and support of gap closing. Sequences of all large repeat elements (rRNA operons and prophages) were confirmed by primer walking of fosmid clones.

The nucleotide sequence for the complete genome sequence of *L. hongkongensis* HLHK9 was submitted to Genbank under accession number CP001154.

### Genome Annotation

Gene prediction was performed by Glimmer [39] version 3.02, and results post-processed using TICO [40] for improving predictions of translation initiation sites. Automated annotation of the finished sequence was performed by a modified version of AutoFACT [41], supplemented by analysis by InterProScan [42]. Manual curation of annotation results was done with support from the software tool GenDB [43]. In addition, annotation of membrane transport proteins was done by performing BLAST search of all predicted genes against the curated TCDB [44]. Ribosomal RNA genes were annotated using the online RNAmmer service [45]. Putative prophage sequences were identified using Prophage Finder [46]. Frameshift errors were predicted using ProFED [47]. CRISPRs (Clustered Regularly Interspaced Short Palindromic Repeats) were searched by using PILER-CR [48], CRISPRFinder [49] and CRT (CRISPR recognition tool) [50].

### Proteomic Analysis by 2D Gel Electrophoresis

Single colony of *L. hongkongensis* HLHK9 was inoculated into brain heart infusion (BHI) medium for 16 h. The bacterial cultures were diluted 1∶100 in BHI medium and growth was continued at 20°C for 20 h and 37°C for 6 h, respectively, with shaking to OD_600_ of 0.6. After centrifugation at 6,500×*g* for 15 min, cells were lysed in a sample buffer containing 7 M urea, 2 M thiourea and 4% CHAPS. The crude cell homogenate was sonicated and centrifuged at 16,000×*g* for 20 min. Immobilized pH gradient (IPG) strips (Bio-Rad Laboratories) (17 cm) with pH 4–7 and 7–10 were hydrated overnight in rehydration buffer containing 7 M urea, 2 M thiourea, 4% CHAPS, 1% IPG buffer pH 4–7 (IPG strip of pH 4–7) and pH 6–11 (IPG strip of pH 7–10) (GE Healthcare) and 60 mM DTT with 60 µg of total protein. The first dimension, isoelectric focusing (IEF), was carried out in a Protean IEF cell electrophoresis unit (Bio-Rad Laboratories) for about 100,000 volt-hours. Protein separation in the second dimension was performed in 12% SDS-PAGE utilizing the Bio-Rad Protean II xi unit (Bio-Rad Laboratories). 2D gels were stained with silver and colloidal Coomassie blue G-250 respectively for qualitative and quantitative analysis, and scanned with ImageScanner (GE Healthcare). ImageMaster 2D Platinum 6.0 (GE Healthcare) was used for image analysis. For MALDI-TOF MS analysis, protein spots were manually excised from gels and subjected to in-situ digestion with trypsin, and peptides generated were analyzed using a 4800 Plus MALDI TOF/TOF Analyzer (Applied Biosystems). Proteins were identified by peptide mass fingerprinting using the MS-Fit software (http://prospector.ucsf.edu) and an in-house sequence database of *L. hongkongensis* HLHK9 proteins generated using the information obtained from the complete genome sequence and annotation. Only spots with at least two-fold difference in their spot volume between 20°C and 37°C and those uniquely detected at either temperature were subjected to protein identification by MALDI-TOF MS analysis. Three independent experiments for each growth condition were performed.

### Essentiality of Arginine for Growth of *L. hongkongensis* HLHK9

*L. hongkongensis* HLHK9 cells were grown in minimal medium M63 [51] supplemented with 20 mM L-malate as carbon source and 19 mM potassium nitrate as nitrogen source, and 1 mM each of vitamin B1 and vitamin B12. The pH of all media was adjusted to 7.0 with KOH. Essentiality of arginine for growth of *L. hongkongensis* HLHK9 was determined by transferring the bacterial cells to the modified M63 medium with or without 100 mM of L-arginine. *Escherichia coli* ATCC 25922 and JW5553-1 (*argB* deletion mutant) [52] were used as positive and negative controls respectively. All cultures were incubated at 37°C with shaking for 5 days. Growth in each medium was determined by measuring absorbance spectrophotometrically at OD_600_. The experiment was performed in duplicate.

### Real-Time RT-PCR

mRNA levels of *argB*-20 and *argB*-37 in *L. hongkongensis* HLHK9 cells grown in 20°C and 37°C were compared. Total RNA was extracted from culture of *L. hongkongensis* HLHK9 (OD_600_ of 0.6) grown in conditions described in proteomic analysis by using RNeasy kit (Qiagen) in combination with RNAprotect Bacteria Reagent (Qiagen) as described by the manufacturer. Genomic DNA was removed by DNase digestion using RNase-free DNase I (Roche). The total nucleic acid concentration and purity were estimated using *A*_260_ */A*_280_ values measured by NanoDrop ND-1000 spectrophotometer (NanoDrop Technologies). Bacteria were harvested from three independent replicate cultures. cDNA was synthesized by RT using random hexamers and SuperScript III kit (Invitrogen) as described previously [53],[54]. cDNA was amplified by TaqMan PCR Core Reagent kit (Applied Biosystems) in an ABI Prism 7000 Sequence Detection System (Applied Biosystems). Briefly, 2 µl of cDNA was amplified in a 25 µl reaction containing 2.5 µl of 10× TaqMan buffer A, 5.5 mM of MgCl_2_, 0.2 mM of each deoxynucleoside triphosphates (dNTPs), 0.8 µM of each primer, 0.8 µM of gene-specific TaqMan probe with a 5′-[6-carboxyfluorescein (6-FAM)] reporter dye and a 3′-[6-carboxytetramethylrhodamine (TAMRA)] quencher dye, 2.5 U of AmpErase Uracil N-glycosylase (UNG) and 0.625 U AmpliTaq Gold polymerase (Applied Biosystems). Primers and TaqMan probes were designed using Primer Express software, version 2.0 (Applied Biosystems) (Table S4). Reactions were first incubated at 50°C for 2 min, followed by 95°C for 10 min in duplicate wells. Reactions were then thermal-cycled in 40 cycles of 95°C for 15 s and 60°C for 1 min. Absolute standard curve method was used for determination of transcript level for each gene. Standard curves were made by using serial dilutions from plasmids containing the target sequences with known quantities. Housekeeping gene RNA polymerase beta subunit, *rpoB*, was used as an internal control. Triplicate assays using RNAs extracted in three independent experiments confirmed that transcript levels of *rpoB* were not significantly different (*P*>0.05) at 20°C compared with 37°C (data not shown). The transcript levels of *argB*-20 and *argB*-37 were then normalized to that of *rpoB*. Triplicate assays using RNAs extracted in three independent experiments were performed for each target gene.

### Phylogenetic Characterization

The phylogenetic relationships among NAGK-20 and NAGK-37 of *L. hongkongensis* HLHK9 and their homologues in other bacteria with complete genomes available were analyzed. Phylogenetic tree was constructed by the neighbor-joining method using Kimura's two-parameter correction with ClustalX 1.83. Three hundred and eleven positions were included in the analysis.

### Cloning and Purification of (His)_6_-Tagged Recombinant NAGK Proteins of *L. hongkongensis* HLHK9

Cloning and purification of (His)_6_-tagged recombinant NAGK proteins of *L. hongkongensis* HLHK9 was performed according to our previous publications, with modifications [53],[55]. To produce plasmids for protein purification, primers (5′- GGAATTCCATATGCTGCTTGCAGACGCCC -3′ and 5′- GGAATTCCATATGTCAGGCTGCGCGGATCAT -3′ for *argB*-20 and 5′- GGAATTCCATATGGTTATTCAATCTGAAGT -3′ and 5′- GGAATTCCATATGTCAGAGCGTGGTACAGAT -3′ for *argB*-37) were used to amplify the genes encoding NAGK-20 and NAGK-37, respectively, by PCR. The sequence coding for amino acid residues of the complete NAGK-20 and NAGK-37 was amplified and cloned, respectively, into the *Nde*I site of expression vector pET-28b(+) (Novagen) in frame and downstream of the series of six histidine residues. The two recombinant NAGK proteins were expressed and purified using the Ni^2+^-loaded HiTrap Chelating System according to the manufacturer's instructions (GE Healthcare).

### Enzyme Assays

Purified NAGK-20 and NAGK-37 were assayed for *N*-acetyl-L-glutamate kinase activity using Haas and Leisinger's method [56], with modifications. The reaction mixtures contained 400 mM NH_2_OH⋅HCl, 400 mM Tris⋅HCl, 40 mM *N*-acetyl-L-glutamate, 20 mM MgCl_2_, 10 mM ATP and 2 µg of enzyme in a final volume of 1.0 ml at pH 7.0. After incubation at 25°C, 30°C, 37°C, 45°C, 50°C, 55°C or 60°C for 30 min, the reaction was terminated by adding 1.0 ml of a stop solution containing 5% (w/v) FeCl_3_⋅6H_2_O, 8% (w/v) trichloroacetic acid and 0.3 M HCl. The absorbance of the hydroxamate⋅Fe^3+^ complex was measured with a spectrophotometer at *A*_540_ [57]. Inhibition of the kinase activities of NAGK-20 and NAGK-37 were examined with and without 0.25, 0.5, 0.75, 1, 2.5, 5, 10, and 20 mM of L-arginine and incubated at 37°C for 30 min. One unit of *N*-acetyl-L-glutamate kinase is defined as the amount of enzyme required to catalyze the formation of 1 µmol of product per min under the assay conditions used. Each assay was performed in duplicate. Results were presented as means and standard deviations of three independent experiments.

## Supporting Information

Figure S1 Physical map of the chemotaxis-related genes in *L. hongkongensis*. While the three gene clusters contain the transducer proteins and some of the methyl-accepting proteins (MCPs), most MCPs are scattered outside the clusters. Genes in orange are coding for chemotaxis transducer proteins; genes in green are coding for MCPs; genes in grey are coding for hypothetical proteins. The numbers refer to the coding sequences in the *L. hongkongensis* genome.
(1.62 MB TIF)

Figure S2 Physical map of six gene clusters of flagellar genes of *L. hongkongensis*. The numbers refer to the coding sequences in the *L. hongkongensis* genome. Genes in pink are regulatory genes for flagellar gene expression; genes in light blue are coding for export apparatus proteins; genes in red are coding for proteins of motor complex/basal body; genes in yellow are coding for hook proteins; genes in green are coding for filament proteins; genes in grey are coding for hypothetical proteins or proteins with other functions; the gene in orange is coding for chemotaxis-related protein.
(1.77 MB TIF)

Figure S3 Phylogenetic analysis of the structural (UreA, UreB and UreC) and accessory proteins (UreE, UreF, UreG, UreD and UreI) in the urease cassette of *L. hongkongensis*. The trees were constructed by the neighbor-joining method and bootstrap values calculated from 1,000 trees. One hundred, 131, 572, 190, 231, 211, 317, and 330 amino acid positions in UreA, UreB, UreC, UreE, UreF, UreG, UreD and UreI, respectively, were included in the analysis. The corresponding amino acid sequences of *S. aureus* were used as outgroups. The scale bar indicates the estimated number of substitutions per 20 or 50 amino acids as indicated. All names and accession numbers are given as cited in the GenBank database.
(0.19 MB PDF)

Figure S4 Schematic diagram of the putative heptosyltransferase and adhesin in *L. hongkongensis* and the corresponding homologues in enterotoxigenic *E. coli* (ETEC). The three functional domains of the putative adhesin are depicted [SS = N-terminal signal sequence (amino acid residues 1–36), a passenger or α-domain (amino acid residues 37–756), translocation or β-domain (amino acid residues 757–1023)]. Alignment of amino acid sequences of the passenger domain of the putative adhesin in *L. hongkongensis* and that of TibA adhesin in ETEC. Residues that match the putative acceptor sites for the heptosyltransferase are boxed. The shaded boxes represent the consensus motifs (Y/V/I/F/W)-X-(F/W) at the last three residues of the translocation domains.
(0.11 MB TIF)

Table S1 Comparison of metabolic pathways for carbohydrate metabolism deduced from the genomes of *L. hongkongensis, C. violaceum, N. gonorrhoeae* and *N. meningitidis*.
(0.03 MB DOC)

Table S2 Comparison of metabolic pathways for amino acid metabolism deduced from the genomes of *L. hongkongensis, C. violaceum, N. gonorrhoeae* and *N. meningitidis*.
(0.04 MB DOC)

Table S3 Comparison of metabolic pathways for fatty acid metabolism deduced from the genomes of *L. hongkongensis, C. violaceum, N. gonorrhoeae* and *N. meningitidis*.
(0.03 MB DOC)

Table S4 Primers and probes for quantitative RT-PCR.
(0.03 MB DOC)

## References

[1] Woo PC, Lau SK, Teng JL, Yuen KY (2005). Current status and future directions of *Laribacter hongkongensis*, a novel bacterium associated with gastroenteritis and traveller's diarrhoea.. Curr Opin Infect Dis.

[2] Yuen KY, Woo PC, Teng JL, Leung KW, Wong MK (2001). *Laribacter hongkongensis* gen. nov., sp. nov., a novel Gram-negative bacterium isolated from a cirrhotic patient with bacteremia and empyema.. J Clin Microbiol.

[3] Lau SK, Woo PC, Hui WT, Li MW, Teng JL (2003). Use of cefoperazone MacConkey agar for selective isolation of *Laribacter hongkongensis*.. J Clin Microbiol.

[4] Woo PC, Lau SK, Teng JL, Que TL, Yung RW (2004). Association of *Laribacter hongkongensis* in community-acquired human gastroenteritis with travel and with eating fish: a multicentre case-control study.. Lancet.

[5] Ni XP, Ren SH, Sun JR, Xiang HQ, Gao Y (2007). *Laribacter hongkongensis* isolated from a community-acquired gastroenteritis in Hangzhou City.. J Clin Microbiol.

[6] Woo PC, Kuhnert P, Burnens AP, Teng JL, Lau SK (2003). *Laribacter hongkongensis*: a potential cause of infectious diarrhea.. Diagn Microbiol Infect Dis.

[7] Lau SK, Woo PC, Fan RY, Lee RC, Teng JL (2007). Seasonal and tissue distribution of *Laribacter hongkongensis*, a novel bacterium associated with gastroenteritis, in retail freshwater fish in Hong Kong.. Int J Food Microbiol.

[8] Teng JL, Woo PC, Ma SS, Sit TH, Ng LT (2005). Ecoepidemiology of *Laribacter hongkongensis*, a novel bacterium associated with gastroenteritis.. J Clin Microbiol.

[9] Lau SK, Woo PC, Fan RY, Ma SS, Hui WT (2007). Isolation of *Laribacter hongkongensis*, a novel bacterium associated with gastroenteritis, from drinking water reservoirs in Hong Kong.. J Appl Microbiol.

[10] Woo PC, Teng JL, Tsang AK, Tse H, Tsang VY (in press). Development of a multi-locus sequence typing scheme for *Laribacter hongkongensis*, a novel bacterium associated with freshwater fish-borne gastroenteritis and traveler's diarrhea.. BMC Microbiol.

[11] Tettelin H, Saunders NJ, Heidelberg J, Jeffries AC, Nelson KE (2000). Complete genome sequence of *Neisseria meningitidis* serogroup B strain MC58.. Science.

[12] Chung GT, Yoo JS, Oh HB, Lee YS, Cha SH (2008). The Complete Genome Sequence of *Neisseria gonorrhoeae* NCCP11945.. J Bacteriol.

[13] Brazilian National Genome Project Consortium (2003). The complete genome sequence of *Chromobacterium violaceum* reveals remarkable and exploitable bacterial adaptability.. Proc Natl Acad Sci U S A.

[14] Lau SK, Li MW, Wong GK, Woo PC, Yuen KY (2008). Distribution and molecular characterization of tetracycline resistance in *Laribacter hongkongensis*.. J Antimicrob Chemother.

[15] Woo PC, Ma SS, Teng JL, Li MW, Kao RY (2005). Construction of an inducible expression shuttle vector for *Laribacter hongkongensis*, a novel bacterium associated with gastroenteritis.. FEMS Microbiol Lett.

[16] Lau SK, Ho PL, Li MW, Tsoi HW, Yung RW (2005). Cloning and characterization of a chromosomal class C β-lactamase and its regulatory gene in *Laribacter hongkongensis*.. Antimicrob Agents Chemother.

[17] Woo PC, Ma SS, Teng JL, Li MW, Lau SK (2007). Plasmid profile and construction of a small shuttle vector in *Laribacter hongkongensis*.. Biotechnol Lett.

[18] Parkhill J, Wren BW, Mungall K, Ketley JM, Churcher C (2000). The genome sequence of the food-borne pathogen *Campylobacter jejuni* reveals hypervariable sequences.. Nature.

[19] Parkhill J, Sebaihia M, Preston A, Murphy LD, Thomson N (2003). Comparative analysis of the genome sequences of *Bordetella pertussis*, *Bordetella parapertussis* and *Bordetella bronchiseptica*.. Nat Genet.

[20] Nikaido H (1996). Multidrug efflux pumps of gram-negative bacteria.. J Bacteriol.

[21] Sperandio V, Torres AG, Kaper JB (2002). Quorum sensing *Escherichia coli* regulators B and C (QseBC): a novel two-component regulatory system involved in the regulation of flagella and motility by quorum sensing in *E. coli*.. Mol Microbiol.

[22] Sperandio V, Mellies JL, Nguyen W, Shin S, Kaper JB (1999). Quorum sensing controls expression of the type III secretion gene transcription and protein secretion in enterohemorrhagic and enteropathogenic *Escherichia coli*.. Proc Natl Acad Sci U S A.

[23] Walters M, Sircili MP, Sperandio V (2006). AI-3 synthesis is not dependent on *luxS* in *Escherichia coli*.. J Bacteriol.

[24] Foster JW (2004). *Escherichia coli* acid resistance: tales of an amateur acidophile.. Nat Rev Microbiol.

[25] Gajiwala KS, Burley SK (2000). HDEA, a periplasmic protein that supports acid resistance in pathogenic enteric bacteria.. J Mol Biol.

[26] Gruening P, Fulde M, Valentin-Weigand P, Goethe R (2006). Structure, regulation, and putative function of the arginine deiminase system of *Streptococcus suis*.. J Bacteriol.

[27] Marquis RE, Bender GR, Murray DR, Wong A (1987). Arginine deiminase system and bacterial adaptation to acid environments.. Appl Environ Microbiol.

[28] Thanassi DG, Cheng LW, Nikaido H (1997). Active efflux of bile salts by *Escherichia coli*.. J Bacteriol.

[29] Prouty AM, Van Velkinburgh JC, Gunn JS (2002). *Salmonella enterica* serovar Typhimurium resistance to bile: identification and characterization of the *tolQRA* cluster.. J Bacteriol.

[30] Benz I, Schmidt MA (1992). Isolation and serologic characterization of AIDA-I, the adhesin mediating the diffuse adherence phenotype of the diarrhea-associated *Escherichia coli strain* 2787 (O126∶H27).. Infect Immun.

[31] Lindenthal C, Elsinghorst EA (2001). Enterotoxigenic *Escherichia coli* TibA glycoprotein adheres to human intestine epithelial cells.. Infect Immun.

[32] Lally ET, Hill RB, Kieba IR, Korostoff J (1999). The interaction between RTX toxins and target cells.. Trends Microbiol.

[33] Banerji S, Flieger A (2004). Patatin-like proteins: a new family of lipolytic enzymes present in bacteria?. Microbiology.

[34] Fernández-Murga ML, Gil-Ortiz F, Llácer JL, Rubio V (2004). Arginine biosynthesis in *Thermotoga maritima*: characterization of the arginine-sensitive *N*-acetyl-L-glutamate kinase.. J Bacteriol.

[35] Caldovic L, Tuchman M (2003). *N*-acetylglutamate and its changing role through evolution.. Biochem J.

[36] Cunin R, Glansdorff N, Piérard A, Stalon V (1986). Biosynthesis and metabolism of arginine in bacteria.. Microbiol Rev.

[37] Haas D, Leisinger T (1975). *N*-acetylglutamate 5-phosphotransferase of *Pseudomonas aeruginosa*. Catalytic and regulatory properties.. Eur J Biochem.

[38] Bartels D, Kespohl S, Albaum S, Drüke T, Goesmann A (2005). BACCardI-a tool for the validation of genomic assemblies, assisting genome finishing and intergenome comparison.. Bioinformatics.

[39] Delcher AL, Bratke KA, Powers EC, Salzberg SL (2007). Identifying bacterial genes and endosymbiont DNA with Glimmer.. Bioinformatics.

[40] Tech M, Pfeifer N, Morgenstern B, Meinicke P (2005). TICO: a tool for improving predictions of prokaryotic translation initiation sites.. Bioinformatics.

[41] Koski LB, Gray MW, Lang BF, Burger G (2005). AutoFACT: an automatic functional annotation and classification tool.. BMC Bioinformatics.

[42] Quevillon E, Silventoinen V, Pillai S, Harte N, Mulder N (2005). InterProScan: protein domains identifier.. Nucleic Acids Res.

[43] Meyer F, Goesmann A, McHardy AC, Bartels D, Bekel T (2003). GenDB-an open source genome annotation system for prokaryote genomes.. Nucleic Acids Res.

[44] Saier MH, Tran CV, Barabote RD (2006). TCDB: the Transporter Classification Database for membrane transport protein analyses and information.. Nucleic Acids Res.

[45] Lagesen K, Hallin P, Rødland EA, Staerfeldt HH, Rognes T (2007). RNAmmer: consistent and rapid annotation of ribosomal RNA genes.. Nucleic Acids Res.

[46] Bose M, Barber RD (2006). Prophage Finder: a prophage loci prediction tool for prokaryotic genome sequences.. In Silico Biol.

[47] Medigue C, Rose M, Viari A, Danchin A (1999). Detecting and analyzing DNA sequencing errors: toward a higher quality of the *Bacillus subtilis* genome sequence.. Genome Res.

[48] Edgar RC (2007). PILER-CR: fast and accurate identification of CRISPR repeats.. BMC Bioinformatics.

[49] Grissa I, Vergnaud G, Pourcel C (2007). CRISPRFinder: a web tool to identify clustered regularly interspaced short palindromic repeats.. Nucleic Acids Res.

[50] Bland C, Ramsey TL, Sabree F, Lowe M, Brown K (2007). CRISPR recognition tool (CRT): a tool for automatic detection of clustered regularly interspaced palindromic repeats.. BMC Bioinformatics.

[51] Miller JH (1972). Experiments in Molecular Genetics.

[52] Baba T, Ara T, Hasegawa M, Takai Y, Okumura Y (2006). Construction of *Escherichia coli* K-12 in-frame, single-gene knockout mutants: the Keio collection.. Mol Syst Biol.

[53] Woo PC, Lau SK, Chu CM, Chan KH, Tsoi HW (2005). Characterization and complete genome sequence of a novel coronavirus, coronavirus HKU1, from patients with pneumonia.. J Virol.

[54] Woo PC, Lau SK, Tsoi HW, Huang Y, Poon RW (2005). Clinical and molecular epidemiological features of coronavirus HKU1-associated community-acquired pneumonia.. J Infect Dis.

[55] Lau SK, Woo PC, Li KS, Huang Y, Tsoi HW (2005). Severe acute respiratory syndrome coronavirus-like virus in Chinese horseshoe bats.. Proc Natl Acad Sci U S A.

[56] Haas D, Leisinger T (1975). *N*-acetylglutamate 5-phosphotransferase of *Pseudomonas aeruginosa*. Catalytic and regulatory properties.. Eur J Biochem.

[57] Lipmann F, Tuttle LC (1945). A specific micromethod for the determination of acyl phosphates.. J Biol Chem.

