# Supplementary figures and images for: The Complete Genome and Proteome of Laribacter hongkongensis Reveal Potential Mechanisms for Adaptations to Different Temperatures and Habitats
Source: PLoS Genet. 2009 Mar 13;5(3):e1000416. doi: 10.1371/journal.pgen.1000416 (PMC2652115; doi:10.1371/journal.pgen.1000416)

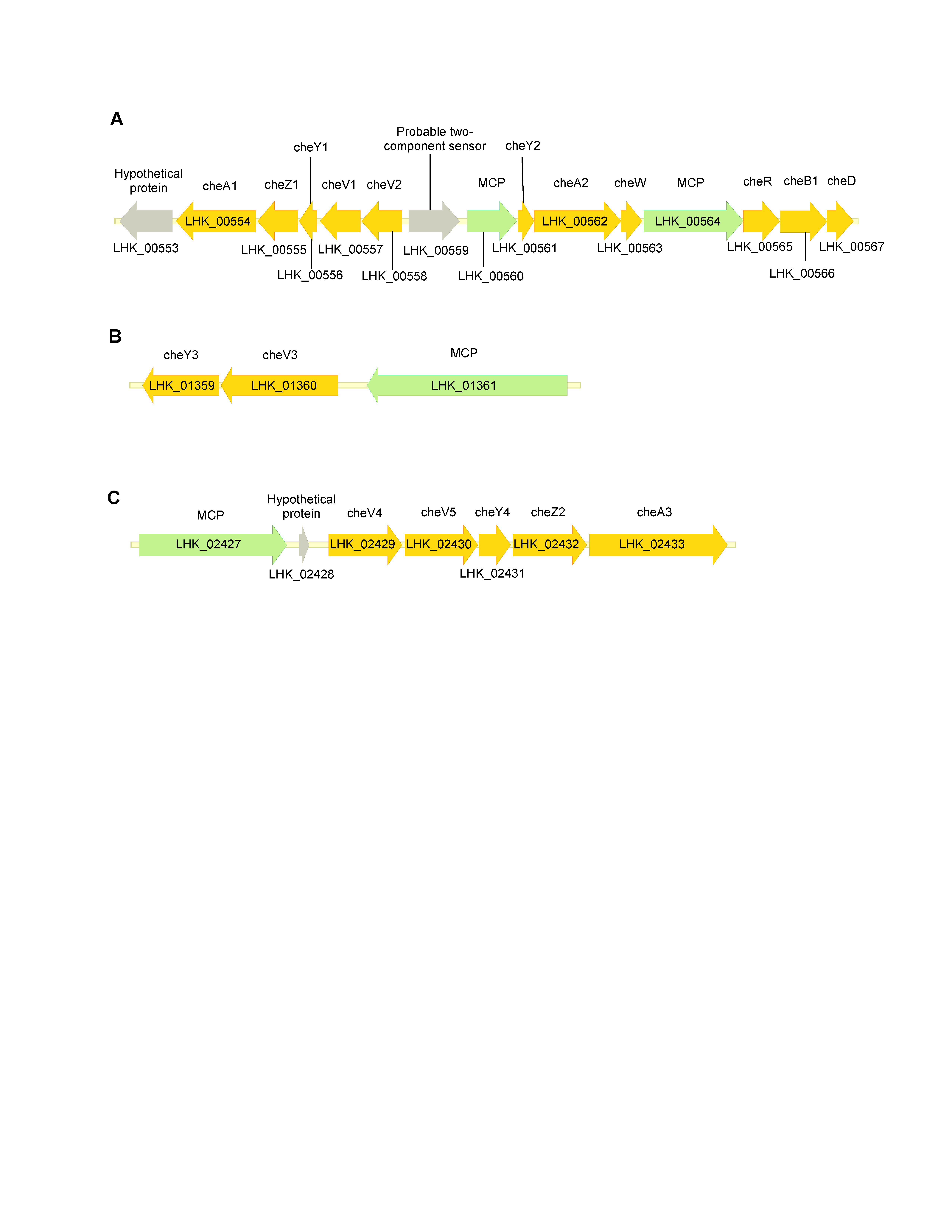

Supplement: Figure S1 — Physical map of the chemotaxis-related genes in L. hongkongensis. While the three gene clusters contain the transducer proteins and some of the methyl-accepting proteins (MCPs), most MCPs are scattered outside the clusters. Genes in orange are coding for chemotaxis transducer proteins; genes in green are coding for MCPs; genes in grey are coding for hypothetical proteins. The numbers refer to the coding sequences in the L. hongkongensis genome. (1.62 MB TIF) [file pgen.1000416.s001.tif]

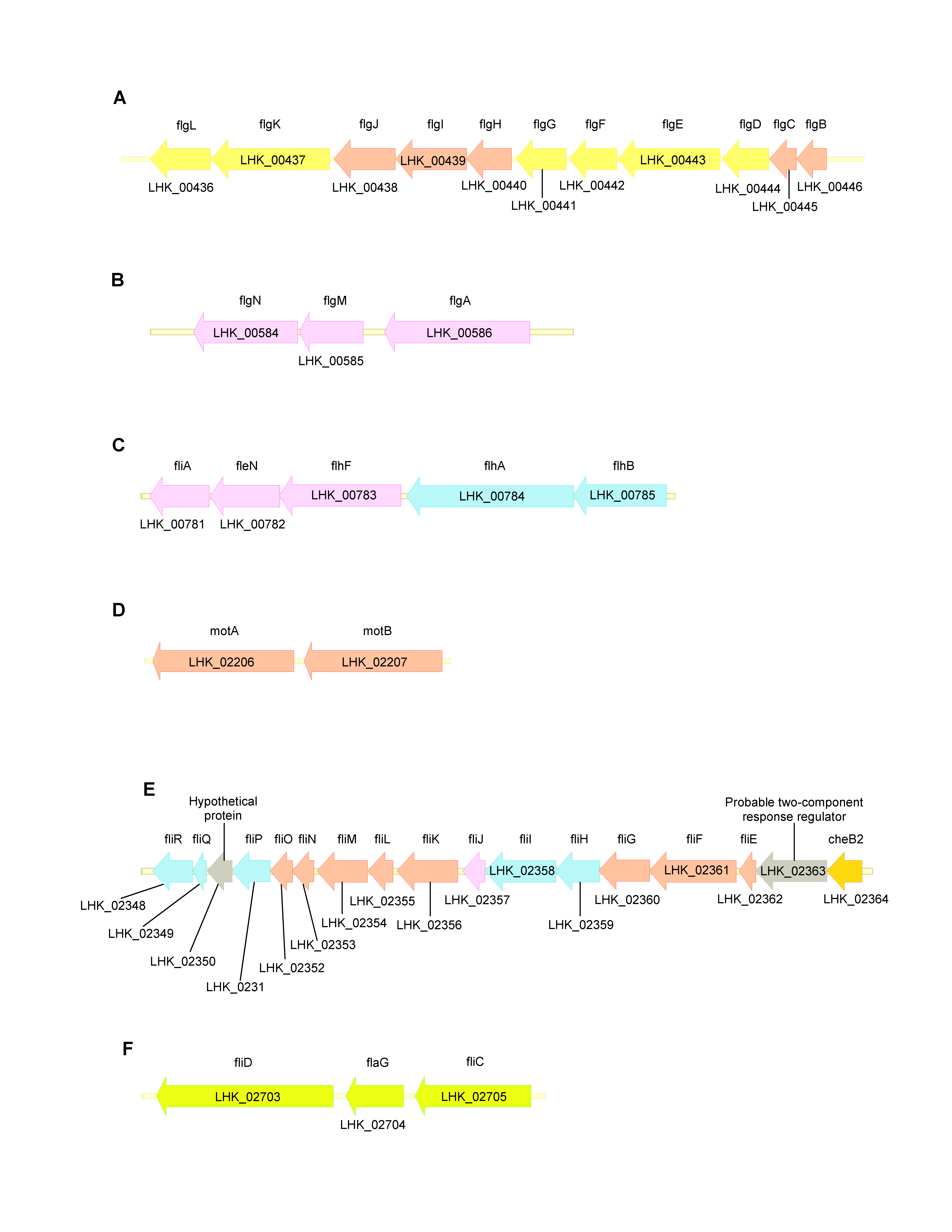

Supplement: Figure S2 — Physical map of six gene clusters of flagellar genes of L. hongkongensis. The numbers refer to the coding sequences in the L. hongkongensis genome. Genes in pink are regulatory genes for flagellar gene expression; genes in light blue are coding for export apparatus proteins; genes in red are coding for proteins of motor complex/basal body; genes in yellow are coding for hook proteins; genes in green are coding for filament proteins; genes in grey are coding for hypothetical proteins or proteins with other functions; the gene in orange is coding for chemotaxis-related protein. (1.77 MB TIF) [file pgen.1000416.s002.tif]

UreA

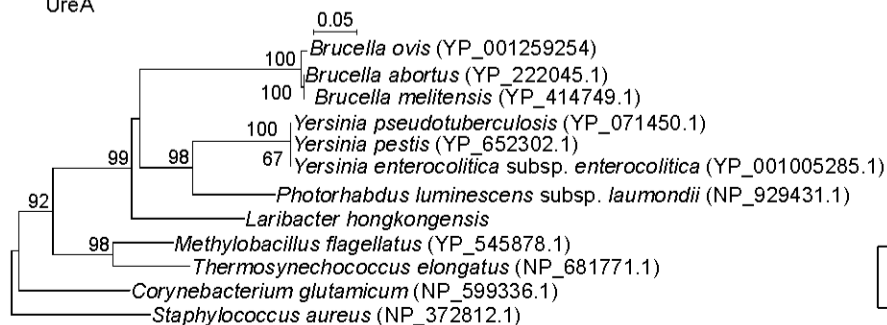

UreB

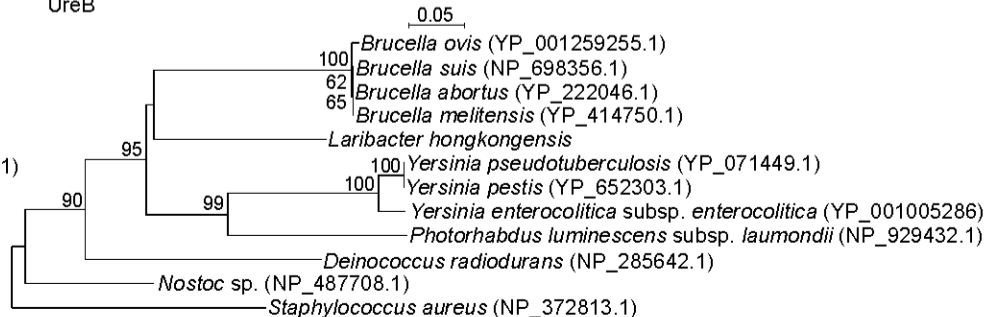

UreC

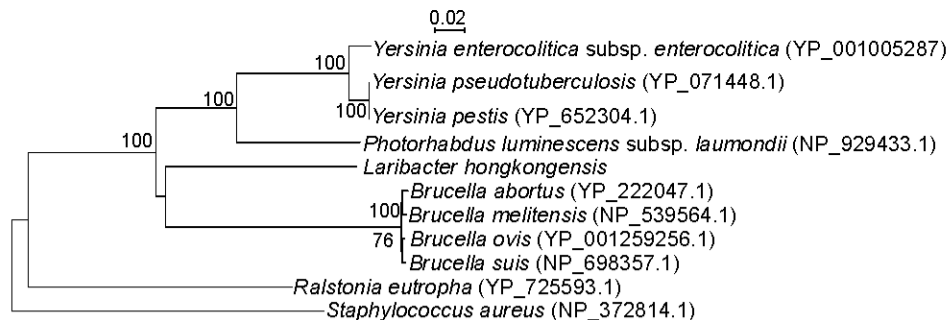

UreE

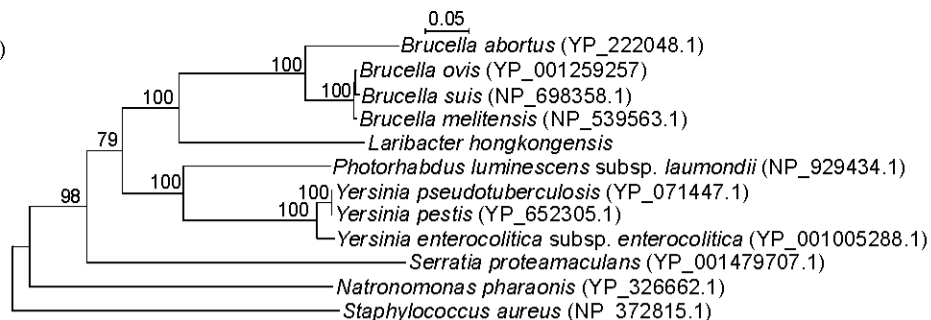

UreF

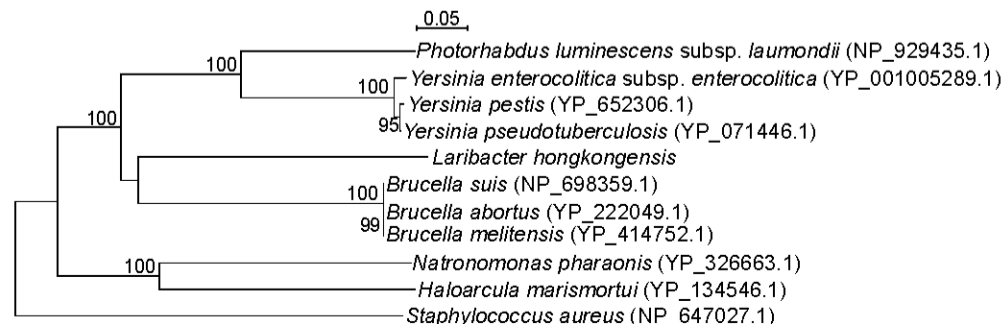

UreG

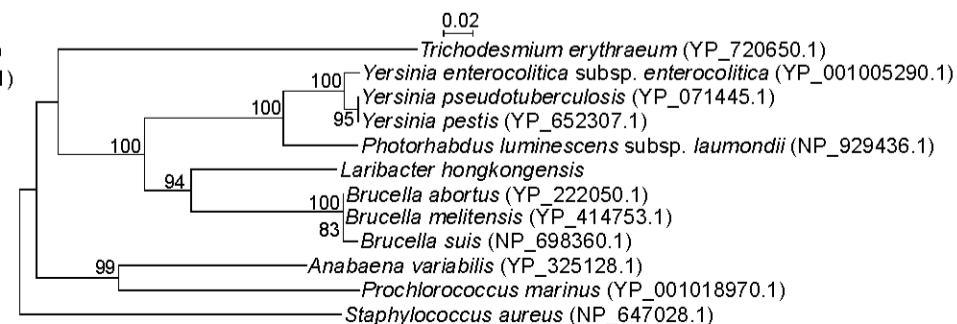

UreD

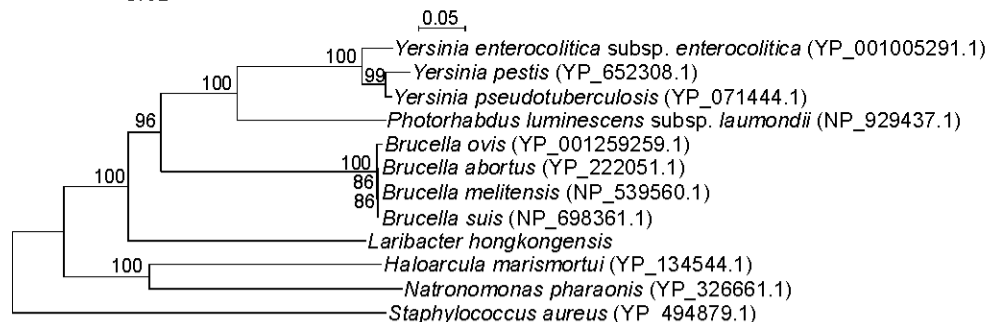

UreI

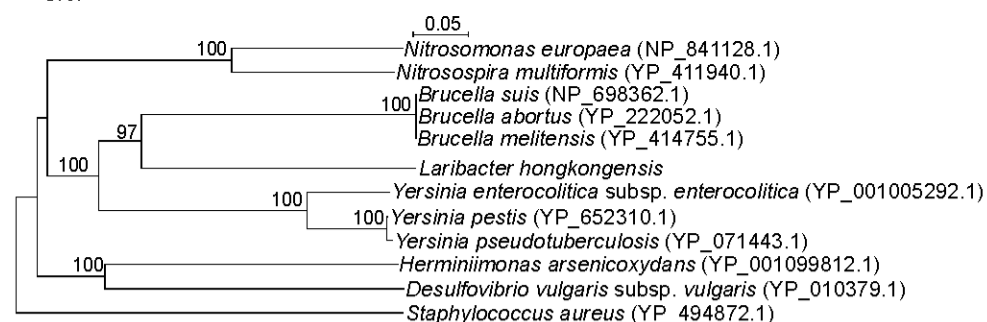

Supplement: Figure S3 — Phylogenetic analysis of the structural (UreA, UreB and UreC) and accessory proteins (UreE, UreF, UreG, UreD and UreI) in the urease cassette of L. hongkongensis. The trees were constructed by the neighbor-joining method and bootstrap values calculated from 1,000 trees. One hundred, 131, 572, 190, 231, 211, 317, and 330 amino acid positions in UreA, UreB, UreC, UreE, UreF, UreG, UreD and UreI, respectively, were included in the analysis. The corresponding amino acid sequences of S. aureus were used as outgroups. The scale bar indicates the estimated number of substitutions per 20 or 50 amino acids as indicated. All names and accession numbers are given as cited in the GenBank database. (0.19 MB PDF) [file pgen.1000416.s003.pdf]

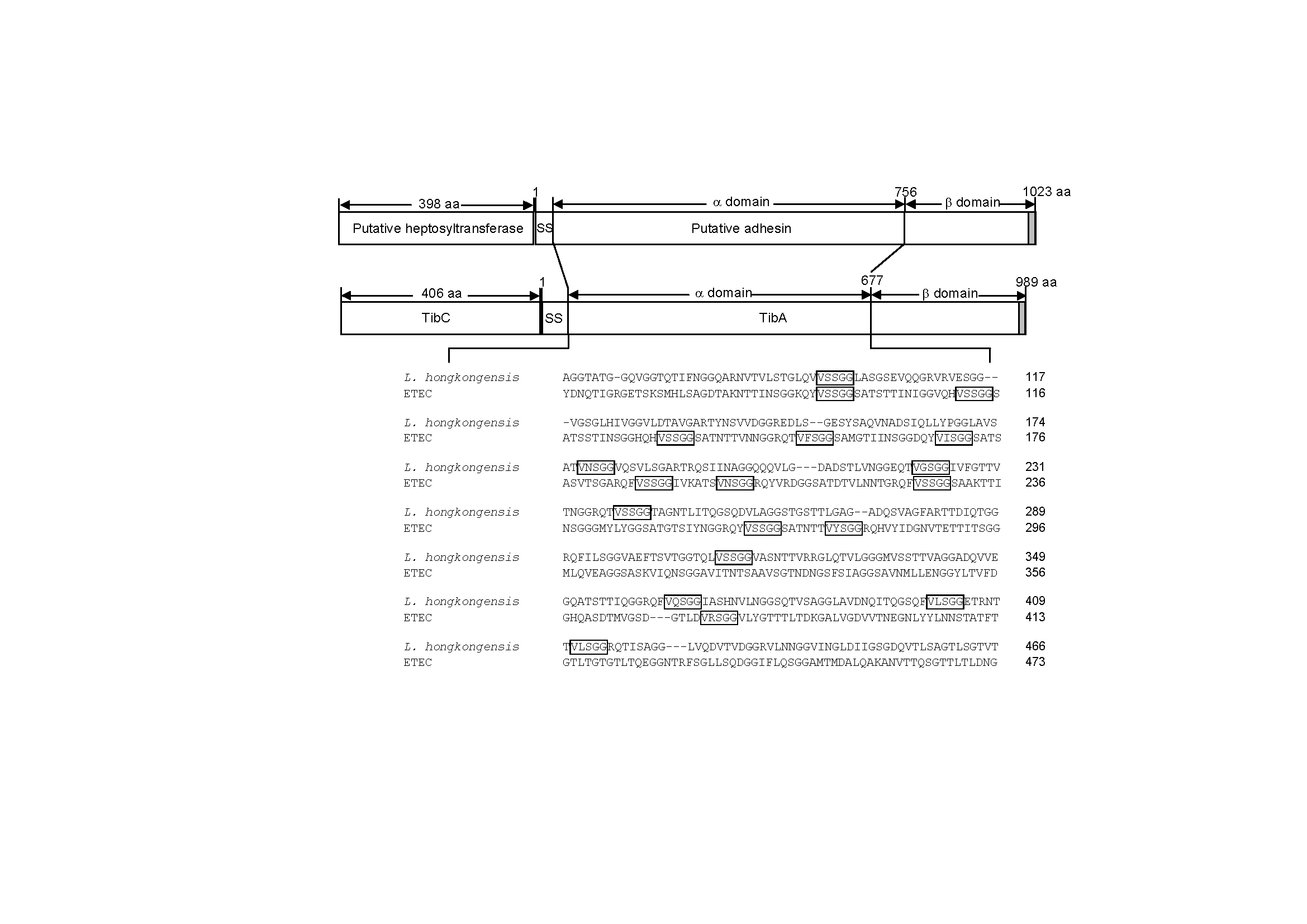

Supplement: Figure S4 — Schematic diagram of the putative heptosyltransferase and adhesin in L. hongkongensis and the corresponding homologues in enterotoxigenic E. coli (ETEC). The three functional domains of the putative adhesin are depicted [SS = N-terminal signal sequence (amino acid residues 1–36), a passenger or α-domain (amino acid residues 37–756), translocation or β-domain (amino acid residues 757–1023)]. Alignment of amino acid sequences of the passenger domain of the putative adhesin in L. hongkongensis and that of TibA adhesin in ETEC. Residues that match the putative acceptor sites for the heptosyltransferase are boxed. The shaded boxes represent the consensus motifs (Y/V/I/F/W)-X-(F/W) at the last three residues of the translocation domains. (0.11 MB TIF) [file pgen.1000416.s004.tif]
