# Supplementary material for: The Complete Genome and Proteome of Laribacter hongkongensis Reveal Potential Mechanisms for Adaptations to Different Temperatures and Habitats
Source: PLoS Genet. 2009 Mar 13;5(3):e1000416. doi: 10.1371/journal.pgen.1000416 (PMC2652115; doi:10.1371/journal.pgen.1000416)
Supplement: Table S1 — Comparison of metabolic pathways for carbohydrate metabolism deduced from the genomes of L. hongkongensis, C. violaceum, N. gonorrhoeae and N. meningitidis. (0.03 MB DOC) [file pgen.1000416.s005.doc]

**Table S1. Comparison of metabolic pathways for carbohydrate metabolism deduced from the genomes of *L. hongkongensis, C. violaceum, N. gonorrhoeae* and *N. meningitidis.***

| Pathways/enzymes | *L. hongkongensis* | *C. violaceum* | *N. gonorrhoeae* | *N. meningitidis* |
| --- | --- | --- | --- | --- |
| Glycolytic pathway | *-* | *+* | *-* | *-* |
| Entner-duodoroff | *-* | *+* | *+* | *+* |
| Gluconeogenesis | *+* | *+* | *-* | *-* |
| TCA cycle | *+* | *+* | *+* | *+* |
| Pentose phosphate pathway | + | + | + | + |
| Glyoxylate bypass | + | + | - | - |
