# Supplementary material for: The Complete Genome and Proteome of Laribacter hongkongensis Reveal Potential Mechanisms for Adaptations to Different Temperatures and Habitats
Source: PLoS Genet. 2009 Mar 13;5(3):e1000416. doi: 10.1371/journal.pgen.1000416 (PMC2652115; doi:10.1371/journal.pgen.1000416)
Supplement: Table S2 — Comparison of metabolic pathways for amino acid metabolism deduced from the genomes of L. hongkongensis, C. violaceum, N. gonorrhoeae and N. meningitidis. (0.04 MB DOC) [file pgen.1000416.s006.doc]

**Table S2. Comparison of metabolic pathways for amino acid metabolism deduced from the genomes of *L. hongkongensis, C. violaceum, N. gonorrhoeae* and *N. meningitidis.***

| Pathways/enzymes | | *L. hongkongensis* | *C. violaceum* | *N. gonorrhoeae* | *N. meningitidis* |
| --- | --- | --- | --- | --- | --- |
| Amino acid biosynthesis | | All 20 amino acids  + selenocysteine | All 20 amino acids | All 20 amino acids | All 20 amino acids |
| Amino acid degradation | |  |  |  |  |
| Serine | | + | + | + | + |
| Alanine | | + | + | + | + |
| Aspartate | | + | + | + | + |
| Asparagine | | + | + | + | + |
| Glutamine | | + | + | + | + |
| Glutamate | | + | + | + | + |
| Glycine | | + | + | + | + |
| Proline | | + | + | - | + |
| Arginine | | + | + | + | + |
|  | Arginine deiminase pathway | + | + | - | - |
|  | Arginine decarboxylase pathway | + | + | + | + |
| Histidine | | - | + | - | - |
| Tyrosine | | - | + | - | - |
