# Supplementary material for: The Complete Genome and Proteome of Laribacter hongkongensis Reveal Potential Mechanisms for Adaptations to Different Temperatures and Habitats
Source: PLoS Genet. 2009 Mar 13;5(3):e1000416. doi: 10.1371/journal.pgen.1000416 (PMC2652115; doi:10.1371/journal.pgen.1000416)
Supplement: Table S3 — Comparison of metabolic pathways for fatty acid metabolism deduced from the genomes of L. hongkongensis, C. violaceum, N. gonorrhoeae and N. meningitidis. (0.03 MB DOC) [file pgen.1000416.s007.doc]

**Table S3. Comparison of metabolic pathways for fatty acid metabolism deduced from the genomes of *L. hongkongensis, C. violaceum, N. gonorrhoeae* and *N. meningitidis.***

| Pathways/enzymes | *L. hongkongensis* | *C. violaceum* | *N. gonorrhoeae* | *N. meningitidis* |
| --- | --- | --- | --- | --- |
| Fatty acid biosynthesis |  |  |  |  |
| Saturated fatty acids | + | + | + | + |
| Unsaturated fatty acids |  |  |  |  |
| *fabA*-*fabB* | - | - | - | - |
| Desaturase | - | + | - | - |
| Fatty acid catabolism |  |  |  |  |
| Saturated fatty acids | + | + | - | - |
| Unsaturated fatty acids | - | + | - | - |
