# Supplementary material for: The Complete Genome and Proteome of Laribacter hongkongensis Reveal Potential Mechanisms for Adaptations to Different Temperatures and Habitats
Source: PLoS Genet. 2009 Mar 13;5(3):e1000416. doi: 10.1371/journal.pgen.1000416 (PMC2652115; doi:10.1371/journal.pgen.1000416)
Supplement: Table S4 — Primers and probes for quantitative RT-PCR. (0.03 MB DOC) [file pgen.1000416.s008.doc]

**Table S4. Primers and probes for quantitative RT-PCR.**

| Genes | Sequence |
| --- | --- |
| *argB*-37 | Forward primer 5’-AAGTACCCGCTCATCTTCACAAC-3’ |
|  | Reverse primer 5′- GATGTACGGCAGGGCTTCTG -3′ |
|  | Probe 5′-[6-FAM]-AGGACACCGCCGCCATCCTG-[TAMRA]-3′ |
| *argB*-20 | Forward primer 5’-GGGCAAGCAGGGAGAGTTC-3’ |
|  | Reverse primer 5′-GGCTGACGATTTCCTTGTTGA-3′ |
|  | Probe 5′-[6-FAM]-CGCGTGACCGATCCGGAAACC-[TAMRA]-3′ |
| *rpoB* | Forward primer 5’-CCAACCAGAACACCAACATCAA-3’ |
|  | Reverse primer 5′-GATGCACCGTCGGCAATC-3′ |
|  | Probe 5′-[6-FAM]-CGTCCGATCGTGAAGGTCGG-[TAMRA]-3′ |
